# A Three-Dimensional Organoid Model of Primary Breast Cancer to Investigate the Effects of Oncolytic Virotherapy

**DOI:** 10.3389/fmolb.2022.826302

**Published:** 2022-02-11

**Authors:** Mary E. Carter, Andreas D. Hartkopf, Anna Wagner, Léa L. Volmer, Sara Y. Brucker, Susanne Berchtold, Ulrich M. Lauer, André Koch

**Affiliations:** ^1^ Department of Obstetrics and Gynaecology, University of Tuebingen, Tuebingen, Germany; ^2^ Department of Internal Medicine VIII, Medical Oncology and Pneumology, University of Tuebingen, Tuebingen, Germany; ^3^ German Cancer Consortium (DKTK), German Cancer Research Center (DKFZ), Partner Site Tuebingen, Tuebingen, Germany

**Keywords:** oncolytic virus, virotherapy, breast cancer, measles virus, vaccinia virus, organoid cell culture, suicide gene, 5-fluorouracil

## Abstract

**Background:** Although several oncolytic viruses have already been tested in early-stage clinical studies of breast cancer, there is still an urgent need to develop patient-derived experimental systems that mimic the response of breast cancer to oncolytic agents in preparation of testing different oncolytic viruses in clinical trials. We addressed this need by developing a protocol to study the effects of oncolytic viruses in stable organoid cell cultures derived from breast cancer tissue.

**Methods:** We used an established three-dimensional organoid model derived from tissue of 10 patients with primary breast cancer. We developed an experimental protocol for infecting organoid cultures with oncolytic viruses and compared the oncolytic effects of a measles vaccine virus (MeV) and a vaccinia virus (GLV) genetically engineered to express either green fluorescent protein (MeV-GFP) and red fluorescent protein (GLV-0b347), respectively, or a suicide gene encoding a fusion of cytosine deaminase with uracil phosphoribosyltransferase (MeV-SCD and GLV-1h94, respectively), thereby enabling enzymatic conversion of the prodrug 5-fluorocytosine (5-FC) into cytotoxic compounds 5-fluorouracil (5-FU) and 5-fluorouridine monophosphate (5-FUMP).

**Results:** The method demonstrated that all oncolytic viruses significantly inhibited cell viability in organoid cultures derived from breast cancer tissue. The oncolytic effects of the oncolytic viruses expressing suicide genes (MeV-SCD and GLV-1h94) were further enhanced by virus-triggered conversion of the prodrug 5-FC to toxic 5-FU and toxic 5-FUMP.

**Conclusions:** We were able to develop a protocol to assess the effects of two different types of oncolytic viruses in stable organoid cell cultures derived from breast cancer tissue. The greatest oncolytic effects were observed when the oncolytic viruses were engineered to express a suicide gene (MeV-SCD and GLV-1h94) in the presence of the prodrug 5-FC. The model therefore provides a promising *in vitro* method to help further testing and engineering of new generations of virotherapeutic vectors for *in vivo* use.

## Introduction

Breast cancer is the most common cause of cancer-associated death in women aged between 20 and 59 years (Siegel et al., 2021). Despite tremendous advances in breast cancer therapy, approximately 20% of all patients experience metastatic recurrence and such metastatic disease still remains incurable. Therefore, there is still an urgent unmet need for new therapeutic options to either prevent and/or treat metastatic disease (Pardoll, 2012).

Oncolytic viruses are emerging as promising agents for the treatment of cancer because they selectively infect and damage cancerous tissues without causing harm to normal tissue (Russell et al., 2012). They offer an attractive combination of tumor-specific cell lysis coupled with immune stimulation through release of tumor antigens and/or other signals to overcome immunosuppression in the tumor microenvironment. Oncolytic viruses achieve tumor-specific lysis in three different ways (Lawler et al., 2017). Firstly, they can enter cells *via* virus-specific, receptor-mediated mechanisms. Secondly, increased viral replication may be supported by rapid cell division in tumor cells. And thirdly, tumor cells support selective virus replication because they often demonstrate deficits in antiviral type I interferon (IFN) signaling (Lawler et al., 2017).

Typically, viruses exhibit a specific cellular tropism that determines which tissues and/or hosts are preferentially infected, and viruses have evolved mechanisms of host cell selectivity by natural selection to improve penetration into host cells. Research conducted with oncolytic viruses led to the differentiation of oncolytic viruses into two groups based on their ability to infect tumor cells. The first group includes oncolytic viruses with natural or intrinsic anti-neoplastic characteristics, and the second group contains ones that have been genetically modified to enhance tumor-selectivity (Hartkopf et al., 2011).

Measles viruses belong to the family of paramyxoviruses (Udem and Cook, 1984). Oncolytic measles viruses are based on attenuated strains which have been used for vaccine purposes for many years and have an excellent safety profile (Aref et al., 2016). Furthermore, they can be genetically engineered with yeast-derived suicide genes that encode for a fusion gene encoding both cytosine deaminase and uracil phosphoribosyltransferase [called FCU1 (Erbs et al., 2000) or SCD (Lampe et al., 2013)] which expresses a chimeric protein that converts the non-toxic prodrug 5-fluorocytosine (5-FC) into highly cytotoxic compound 5-fluorouracil (5-FU) and subsequently into 5-fluorouridine monophosphate (5-FUMP), thereby bypassing an important mechanism of chemoresistance for 5-FU (Hartkopf et al., 2013). 5-FU is a cytotoxic agent that is used for the treatment of breast cancer, and 5-FUMP is the activated form of 5-FU (Slos and Erbs, 2004; Dias et al., 2010). Cytosine deaminase catalyzes the conversion to 5-FU and uracil phosphoribosyltransferase (UPRT) catalyzes the subsequent conversion of 5-FU to 5-FUMP. Therefore, UPRT has the potential to sensitize chemoresistant cancer cells to 5-FU (Hartkopf et al., 2013). An anti-tumor effect of SCD on cancer cells has already been demonstrated in an adenovirus model (Graepler et al., 2005). Oncolytic measles vaccine virus MeV-SCD has previously demonstrated tumor-specific replication in experiments in human hepatoma and ovarian cancer cells (Hartkopf et al., 2013; Lampe et al., 2013). Several oncolytic measles viruses are undergoing clinical development in cancer patients for a variety of malignant diseases, e.g., ovarian or breast cancer (Galanis et al., 2010; Lech and Russell, 2010).

Vaccinia viruses belong to the poxvirus family. Their oncolytic properties have already been demonstrated in clinical trials, while causing only mild flu-like symptoms (Hunter-Craig et al., 1970; Arakawa et al., 1987; Gomella et al., 2001). Similar to oncolytic measles virus MeV-SCD, oncolytic vaccinia virus GLV-1h94 also, encodes the FCU1 suicide fusion gene enabling enzymatic conversion of 5-FC to 5-FU and 5-FUMP (Slos and Erbs, 2004).

Extensive attempts to develop oncolytic viruses for breast cancer with the previously established methods have not been successful so far. Hence, oncolytic viruses have not yet been approved for the treatment of breast cancer. Previously established models used for experimental cancer research, including two-dimensional cultures of immortalized cell lines, patient-derived xenograft models and transgenic mice, fail to mimic adequately the complex tumor microenvironment of human cancer (Yuki et al., 2020). These models have major disadvantages and may only insufficiently represent the patterns of the original cancer patient tumor tissues (Bosma and Carroll, 1991; Kamb, 2005). In particular, many patient-derived xenograft models of breast cancer do not recapitulate the tumor microenvironment of their tumor origin, have low success rates of tumor transplantation, and are relatively expensive because of the need for immune-deficient mice (Murayama and Gotoh, 2019). A three-dimensional organoid model based on patient-derived tumor samples may offer a better way forward. This model should be able to mimic the tumor-immune interactions and mutational status of the original tumor (Bar-Ephraim et al., 2020; Yuki et al., 2020). Additionally, it offers the future prospect of integrating the individual immune system into the model, thereby increasing the reliability of research to enhance the transition of new therapies from bench to beside (Bar-Ephraim et al., 2020). Currently, the addition of the immune system to patient-derived breast cancer organoid cultures is under investigation by several groups. For example, autologous peripheral blood monocytes derived from patient blood samples have been successfully added into the corresponding colon and lung cancer organoid setup (Dijkstra et al., 2018). Tumor-on- a-chip technology may also enable a better understanding of the role of the immune system and its incorporation into an organoid model setup (Moccia and Haase, 2021).

Accordingly, there is an urgent need to develop patient-derived experimental systems that recreate the different aspects of breast cancer *in vitro* to investigate hallmark parameters such as efficiencies of virotherapeutic infections, kinetics of intratumoral viral replication and immune-mediated oncolysis. Recent advances in three-dimensional cell culture technology enable culture of embryonic and adult mammalian stem cells in a way that allows them to exhibit their self-organizing properties. The resulting organoids mimic important structural and functional properties of different organs such as kidney, lung, intestine, brain and retina and are currently under investigation as models for predicting drug response especially with regard to personalized cancer treatment (Clevers, 2016; Drost and Clevers, 2018; Rosenbluth et al., 2020). For example, an organoid model of pancreatic cancer and healthy pancreatic tissue was used to determine the effects of oncolytic adenoviruses, and the authors concluded that the response of the pancreatic organoid model to oncolytic adenoviruses might be indicative of in-patient responses of primary pancreatic tumors and metastases (Raimondi et al., 2020). The culture conditions for human mammary epithelial organoids have already been established that create organoids which exhibit the histological and genetic features of the original tumors (Sachs et al., 2018).

In this study we set out to answer the question whether three-dimensional cell cultures are suitable for testing oncolytic virotherapy. We addressed this topic by developing a protocol in a stable three-dimensional organoid model derived from patients with primary breast cancer to determine the oncolytic effects of genetically engineered oncolytic viruses, encoding either marker genes for GFP (oncolytic measles virus MeV-GFP) and for red fluorescent protein (oncolytic vaccinia virus GLV-0b347), or the SCD/FCU1 suicide gene (oncolytic measles virus MeV-SCD, oncolytic vaccinia virus GLV-1h94) on breast cancer organoid cultures.

## Methods

### Breast Cancer Patients and Tumor Tissues

Tissue was obtained within a period of 4 months in 2019 from ten female patients aged between 30 and 70 years, who had been diagnosed with primary invasive breast cancer at the obstetrics and gynecology department of the University Hospital Tuebingen (Table 1) and who had not received systemic chemotherapy or radiation. Consequently, patients diagnosed with recurrent breast cancer or metastases were excluded from the study. Infiltration of tumor cells in lymph nodes was not defined as an exclusion criterion.

**TABLE 1:** Tumor characteristics of patients providing breast cancer tissues for the establishment of tumor organoid cultures. ER = estrogen receptor; ER-IRS = estrogen receptor immunoreactive score; PR = progesterone receptor; PR-IRS = progesterone receptor immunoreactive score, Her2 = human epidermal growth factor 2, Her2-IHC-Score = human epidermal growth factor 2-ImmunoHistoChemistry score.

	Age	Diagnosis	Grading	ER		PR		Her2	Her2-IHC-score	Ki67
BC-ORG	37	Invasive ductal carcinoma	G1	Pos.	ER-IRS:12	Pos.	PR-IRS:12	Neg.	1+	10%
1					90% ER-staining		90%			
BC-ORG	49	Invasive lobular carcinoma	G2	Pos.	ER-IRS:12	Pos.	PR-IRS:4	Neg.	1+	5%
2					90% ER-staining		90%			
BC-ORG	52	Invasive ductal carcinoma	G3	Neg.	ER-IRS:0	Neg.	PR-IRS:0	Neg.	1+	60%
3					0% ER staining		2%			
BC-ORG	42	Mucinous with associated ductal carcinoma *in situ*	G2	Pos.	ER-IRS:9	Pos.	PR-IRS:6	Pos.	2+ (FISH pos.)	15%
4					80% ER staining		40%			
BC-ORG	59	Invasive lobular carcinoma with associated lobular carcinoma *in situ*	G2	Pos.	ER-IRS:12	Pos.	PR-IRS:1	Neg.	1+	10%
5					100% ER staining		1–9%			
BC-ORG6	67	Invasive lobular carcinoma	G2	Pos.	ER-IRS:12100% ER staining	Pos.	PR-IRS:6 n.d.	Neg.	0	10–15%
BC-ORG	56	Invasive ductal carcinoma	G2	Pos.	ER-IRS:12	Pos.	PR-IRS:12	Neg.	1+	5%
7					100% ER staining		100%			
BC-ORG	52	Tubular carcinoma	G1	Pos.	ER-IRS:12	Pos.	PR-IRS:6	Neg.	1+	5%
8					90% ER-staining		60%			
BC-ORG	51	Invasive ductal carcinoma	G3	Pos.	ER-IRS:12	Pos.	PR-IRS:1	Pos.	3+	10–15%
9					100% ER-staining		1%			
BC-ORG 10	62	Invasive ductal carcinoma	G2	Pos.	ER-IRS:12	Pos.	PR-IRS:12	Neg.	1+	10–15%
					100% ER-staining		100%			

All of the original tumor specimen included in this study were at least 1 cm^3^ in size to enable full histopathological analysis, yet still allowing the harvest of sufficient numbers of tumor cells for cultivation. All patients provided written informed consent and the study was approved by the local ethics committees (210/2019BO2).

### Processing Breast Cancer Patient Tissue for Establishing Organoid Cell Cultures

**FIGURE 1:**
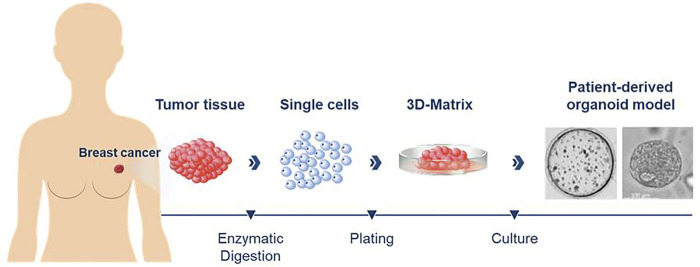
Diagram illustrating the process for preparing patient-derived organoid cultures from breast cancer tissues. Enzymatic digestion of fresh tumor tissues was used to generate singe cells that were then plated in a three-dimensional matrix for culture of the organoids.

Figure 1 illustrates the overall process for preparing breast cancer patient-derived organoid cultures. Tumor tissues derived from each patient were cut into 1 mm^3^ sized pieces and digested with a 1:1 mix of advDMEM/F12 +/+/+ (Gibco Advanced Dulbecco’s Modified Eagle Medium/F-12 with the addition of 1% GlutaMAX, 1% 4-(2-hydroxyethyl)-1-piperazineethanesulfonic acid (HEPES) and 1% penicillin-streptomycin (all reagents from Thermo Fisher Scientific, Waltham, MA), collagenase (type IV 5 mg/ml, Sigma-Aldrich, Munich, Germany) and 10 µM Y-27632 (Hoelzel Diagnostika, Cologne, Germany) until sufficient digestion was achieved (after 1–3 h, indicated by the onset of clouding of the solution). The suspension was then transferred into a 15 ml tube containing 10 ml of advDMEM/F12^+/+^/+ and centrifuged at 478 x g for 10 min. The supernatant was removed and the pellet resuspended in 1 ml of TrypLE Express (Thermo Fisher Scientific) and incubated for another 15–30 min. The solution was filtered through a 100 µm filter into a 50 ml tube and washed with additional 10 ml advDMEM F12^+/+^/+. The suspension was centrifuged for 478 x g for 10 min and the supernatant carefully removed. Depending on the size of the remaining cell pellet, it was resuspended in 60–500 µL of advDMEM/F12 +/+/+. For an organoid setup (6 wells in a 48-well plate) an aliquot of 60 μL cell suspension was mixed with 70 µL of Matrigel (Corning, NY, USA). Aliquots of 20 µL were pipetted into each well of a 48-well plate. Afterwards, the culture plate was placed upside down in an incubator at 37°C and 5% CO_2_. After 30 min, 280 µL of breast cancer culture medium were added to each well. The medium was changed every 3–4 days. The residual cell suspension not used for plating was resuspended in 700 µL Gibco Recovery Cell Culture Medium (Thermo Fisher Scientific) per vial and frozen. The vials were then transferred to −80°C in cell coolers. For long-term storage the vials were transferred to containers containing liquid nitrogen.

### Passaging of Organoid Cultures

The organoid lines were passaged and split based on the confluency of the culture (organoids in the center of the Matrigel drop shedding debris or becoming darker in appearance and/or becoming larger than 300 µm in diameter) ranging from 5–20 days. After removal of the culture medium the wells were washed with 1 ml of PBS. Afterwards the Matrigel domes were mechanically scraped off the bottom of the culture plate with a pipette tip and collected in TrypLE Express (1 ml for 6 wells). The solution was incubated for 5 min at 37°C. After the addition of 10 ml of advDMEM/F12 +/+/+ the solution was centrifuged for 10 min at 478 x g. The cell pellet was resuspended in advDMEM/F12 +/+/+ and organoids were plated and cryopreserved as described earlier.

### Breast Cancer Tissue Organoid Culture Medium

The medium used for culturing the breast cancer organoid cultures contained the following ingredients: 50% conditioned medium from L-WRN cells (ATCC #CRL-3276) (Miyoshi and Stappenbeck, 2013) (containing Wnt3a, R-spondin 3, and Noggin), Heregulin 5 nmol/L (Peprotech, NJ, USA), fibroblast growth factor 7 (FGF7) 5 ng/ml (Peprotech), fibroblast growth factor 10 (FGF10) 20 ng/ml (Peprotech), epidermal growth factor (EGF) 5 ng/ml (Peprotech), A83-01 500 nmol/L (Tocris, Wiesbaden, Germany), Y27632 5 µmol/L (Hölzel), SB202190 (Sigma-Aldrich), Gibco B27 Supplement 2% (Thermo Fisher Scientific), N-acetyl-cysteine 1.25 mmol/L (Sigma-Aldrich), nicotinamide 5 mmol/L (Sigma-Aldrich), Primocin 50 µg/ml (InvivoGen, Toulouse, France), Gibco advDMEM/F12 50% (Thermo Fisher Scientific).

### Infecting Breast Cancer Organoid Cultures With Oncolytic Viruses

#### Protocol 1–Infection With Oncolytic Viruses 24 h After Passaging

Organoids were passaged according to the standard method described above. After centrifugation and removal of the supernatant the cell pellet was resuspended in 260 µL of breast cancer culture medium. Aliquots of 60 µL were taken for plating out in 60% Matrigel according to the standard passaging protocol for further cultivation. For the remaining 200 µL of organoid suspension, 2,500 µL of breast cancer culture medium and 300 µL of Matrigel were added. This suspension was plated into 12 wells of an untreated 48-well cell culture plate with a 250 µL drop size and an estimated density of 2.5 × 10^4^ cells per well. After 24 h the amount of virus calculated for a defined viral concentration was suspended in breast cancer culture medium and 50 µL per well were added. This results in two wells for each concentration point. The plate was then left to remain warm in the incubator at 37°C and 5% CO_2_. Infection state and virus distribution were monitored daily and documented photographically.

#### Protocol 2—Infection With Oncolytic Viruses While Passaging

Organoids were passaged according to the method described above. After centrifugation and removal of the supernatant the cell pellet was resuspended in 300 µL of breast cancer culture medium. Aliquots of 90 µL of this suspension were used for cultivation in 60% Matrigel according to the aforementioned method (passaging of organoids). Aliquots of 10 µL of suspension were used to count in an improved-Neubauer cell counting chamber. The remaining 200 µL of suspension were used for plating the cells in 10% Matrigel. Each well consisted of 225 µL of breast cancer culture medium with the cells/organoids, 25 µL of Matrigel, and 50 µL of breast cancer culture medium with the virus (in case of the control wells additional breast cancer culture medium was used). A total of 18 wells were required for each infection. The 200 µL organoid suspension was resuspended in breast cancer culture medium and Matrigel. From this suspension 500 µL were removed and 100 µL of the desired viral suspension added. Aliquots of 300 µL were plated out into one well at a time, resulting in the desired two wells for each viral concentration. This was repeated for all the desired viral concentrations. An untreated 48-well culture plate was used. The plate was then placed in an incubator at 37°C with 5% CO_2_. The viral distribution was monitored daily through microscopy and photographically documented each day.

#### Protocol 3–Infection of Organoid Cultures With Oncolytic Viruses 7–10 Days After Passaging

Organoid cultures were passaged according to the method described above and plated out in 6 wells of a 48-well culture plate treated with 60% Matrigel. The organoids were placed in a CO_2_ incubator for 7–10 days until the organoid cultures had reached a sufficient size and density for viral infection. Then aliquots of 100 µL of dispase II (1 mg/ml) were added to each well while mechanically scraping the Matrigel dome from the bottom of the well. The cell culture plate was returned to the CO_2_ incubator at 37°C for 60 min. Then the contents of the wells were removed and transferred into a 15 ml tube. The wells were then washed with 1 ml of Dulbecco’s PBS. This was also added to the 15 ml tube and centrifuged at 210 x g for 15 min. The supernatant was carefully removed with a pipette and discarded. The cell pellet was resuspended in 5,625 µL breast cancer culture medium. Aliquots of 625 µL of Matrigel were added. Subsequently, 520 µL of the suspension were pipetted into a 1.5 ml tube and 100 µL of the desired viral concentration were added. Then 300 µL of this suspension were transferred into a well of an untreated 48-well cell culture plate thereby resulting in two wells with the same viral concentration. The cell culture plate was then placed in an incubator at 37°C with 5% CO_2_.

### Viral Titration

We used a cell density of 25,000 cells per well and investigated the effects of MeV-GFP, MeV-SCD, GLV-0b347 and GLV-1h94 (see below). All viruses were used in a concentration termed multiplicity of infection (MOI) equal to 10 (meaning that the ratio of infectious viral particles to tumor cells had been adjusted to 10:1). In addition, MOI 1 was also used for MeV-GFP and MOIs of 0.1 and 1 were also used for GLV-0b347. The prodrug 5-fluorocytosine (5-FC) was added to infections with MeV-SCD and GLV-1h94 at a concentration of 1 mmol/L. Additionally, the following controls were measured for each organoid line: 1) 1 mmol/L 5-FC, 2) MeV-SCD/GLV-1h94 without the prodrug 5-FC and 3) 1 mmol/L 5-FU as well as 4) two wells containing breast cancer culture medium only.

Organoids were dissipated into single cells for counting with an improved-Neubauer cell counting chamber to enable an estimation of the cells seeded out to for organoid growth and viral infection. An aliquot of 10 µL of the organoid suspension used for viral infection was incubated with TrypLE Express for 20 min to allow for dissipation of the organoids into single cells. The solution was then used for cell counting with an improved-Neubauer cell counting chamber. Approximately 25,300 ± 10,300 cells (mean ± SD, N = 10) in the form of organoids were contained in each well.

### Oncolytic Measles Viruses

**FIGURE 2:**
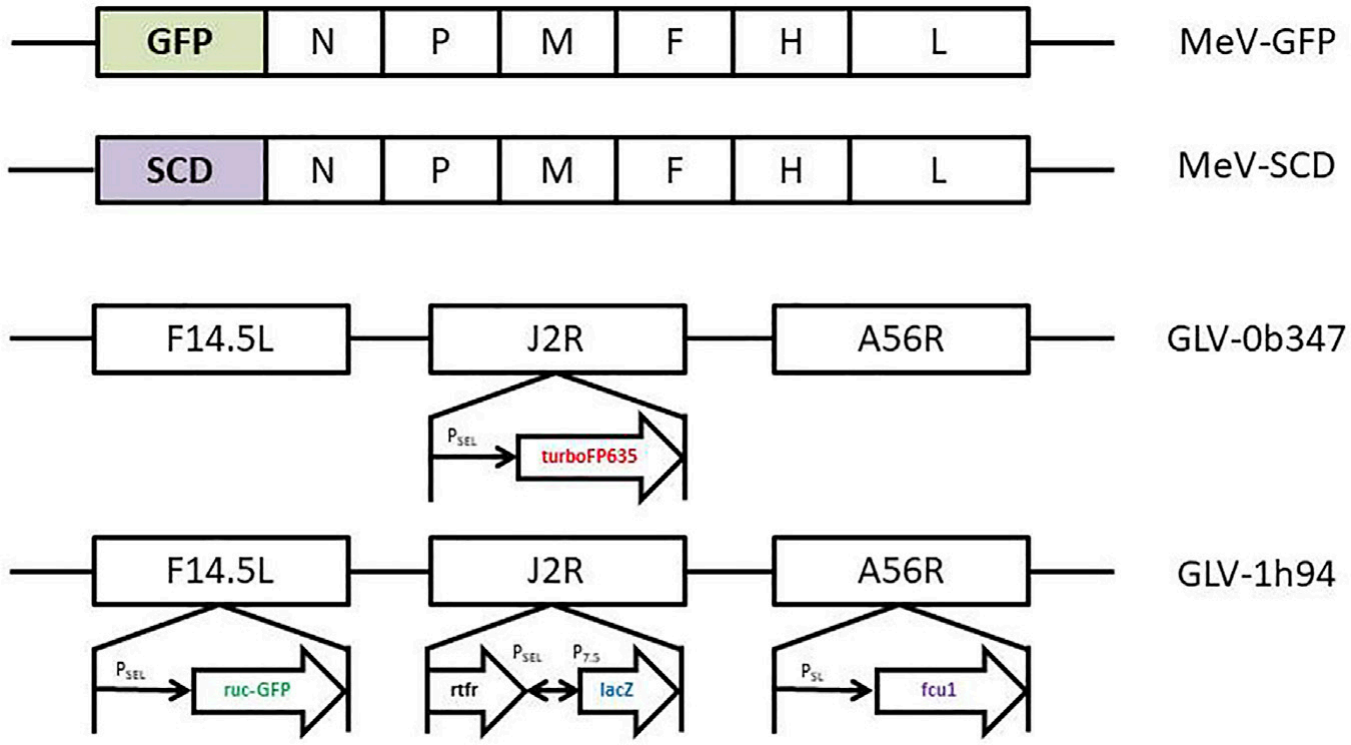
Schematic depiction of the relevant sections of the oncolytic virus genomes for the viruses used in this study. MeV-GFP: The gene encoding for green fluorescent protein (GFP) has been inserted as a transgenic marker gene upstream of the N-gene of the Measles virus genome. MeV-SCD: The gene encoding for the SCD/FCU1 suicide gene has been inserted at the very same position. Key for the MeV genomes: N = nucleocapsid gene; P = phosphoprotein gene; M = matrix protein gene; F = fusion protein gene; H = hemagglutinin gene; L = large protein gene. GLV-0b347: The gene encoding red fluorescent protein (turboFP635) has been inserted in the J2R locus, thus deleting the thymidine kinase function of the respective vaccinia viruses. GLV-1h94: The SCD/FCU1 suicide gene has been inserted in the F14.5L locus. Key to the GLV genomes: F14.5L = open reading frame encoding 49 amino acids; J2R = non-essential gene encoding vaccinia thymidine kinase; A56R = non-essential gene encoding hemagglutinin; turboFP635 = far red mutant of the red fluorescent protein from sea anemone *Entacmaea quadricolor*; ruc-GFP = *Renilla reniformis* luciferase—*Aequorea victoria* green fluorescent fusion protein; rtfr = reverse gene of human transferrin receptor; lacZ = β-galactosidase; P_SEL_ = VACV (vaccinia virus) synthetic early/late promoter; P_SL_ = VACV synthetic late promoter; P_7.5_ = VACV early/late promoter.

Oncolytic measles viruses were genetically modified from the *Schwarz* vaccine strain. MeV-GFP is a live attenuated, recombinant oncolytic measles virus in which the genetic information for the GFP marker protein was integrated at genome position one (Figure 2). MeV-SCD is a live attenuated, recombinant oncolytic measles virus in which the genetic information for the prodrug converting enzyme super cytosine deaminase (SCD; i.e., a fusion protein of yeast cytosine deaminase and uracil phosphoribosyltransferase) was integrated at the same genome position (Figure 2). Expression of GFP allows the monitoring of both viral infection and spread*.*

### Oncolytic Vaccinia Viruses

Vaccinia virus GLV-0b347 is derived from a *Western Reserve* vaccinia virus strain (Figure 2). The locus of the J2R gene, encoding for a thymidine kinase, has been replaced with a vaccinia synthetic early/late promotor and TurboFP635, a red fluorescent protein derived from the sea anemone *Entacmaea quadricolor* (Figure 2) (Wiedenmann et al., 2002). Disruption of J2R moreover results in reduced virulence (Zhang et al., 2009). GLV-1h94 contains a *Lister* vaccinia virus strain (LIVP) backbone (Zhang et al., 2009). In GLV-1h94 the A56R gene (encoding for a thymidine kinase) has been disrupted by the insertion of the vaccinia synthetic early/late promoter and the suicide gene FCU1, also leading to an attenuated virus (Zhang et al., 2007). GLV-1h94 expresses the Renilla luciferase–Aequorea green fluorescent protein (RUC-GFP) expression cassette in the gene locus of F14.5L, resulting in an inactivation of the F14.5L gene. The gene locus F14.5L encodes a protein important for cell adhesion and virulence (Izmailyan and Chang, 2008).

### Fluorescence Microscopy

Starting 24 h after the infection, imaging was performed on all organoid cultures every 24 h to depict viral spread in the breast cancer cells. The microscope (Olympus IX50 inverted fluorescence phase-contrast microscope) used, was permanently connected to an F-view camera system (Soft Imaging System GmbH, Muenster Germany). Pictures taken with phase contrast (100 ms exposure time) and fluorescence (150 ms–5 s exposure time) were processed using AnalySIS version 3.1 software (Soft Imaging System GmbH, Muenster, Germany).

### CellTiter-Blue^®^ Viability Assay

We used the CellTiter-Blue^®^ Assay (Promega, Walldorf, Germany) to measure the viability of organoids after infection. An aliquot of 60 µL was added per well. The plate was then placed back in the incubator for 90 min and measured using a Synergy HT microplate reader and Gen5.11 software (BioTek Instruments, Winooski, VT).

### Statistical Analysis

To determine the percentage of surviving cells with the CellTiter-Blue^®^ Assay we divided the read out of organoids treated with virus, 5-FC or 5-FU by the read out of untreated organoids (no virus, 5-FC or 5-FU). As Matrigel alone exhibits a small signal with the assay, this control value was deducted from all original values before calculating the percentage of surviving cells. All data are expressed as mean ± standard deviation (SD) of ten independent experiments performed in duplicate. Statistical analyses were performed using GraphPad Prism software (GraphPad Holdings, LLC, San Diego, CA, USA). A one-way analysis of variance (ANOVA) was performed to determine whether there were any significant differences between the groups or between the different MOIs of 10, 1 and 0.1. Subsequent post-hoc Tukey’s multiple comparisons tests were performed to determine statistical significance between any two groups. A significance level of *p* < 0.05 was used to reject the null hypothesis that there was no difference between the groups tested. We expressed the level of significance with the following annotations in the figures: *p < 0.05, **p < 0.01, ***p < 0.001, and ****p < 0.0001.

## Results

### Stable Organoid Cultures Prepared From Breast Cancer Tissues

**FIGURE 3:**
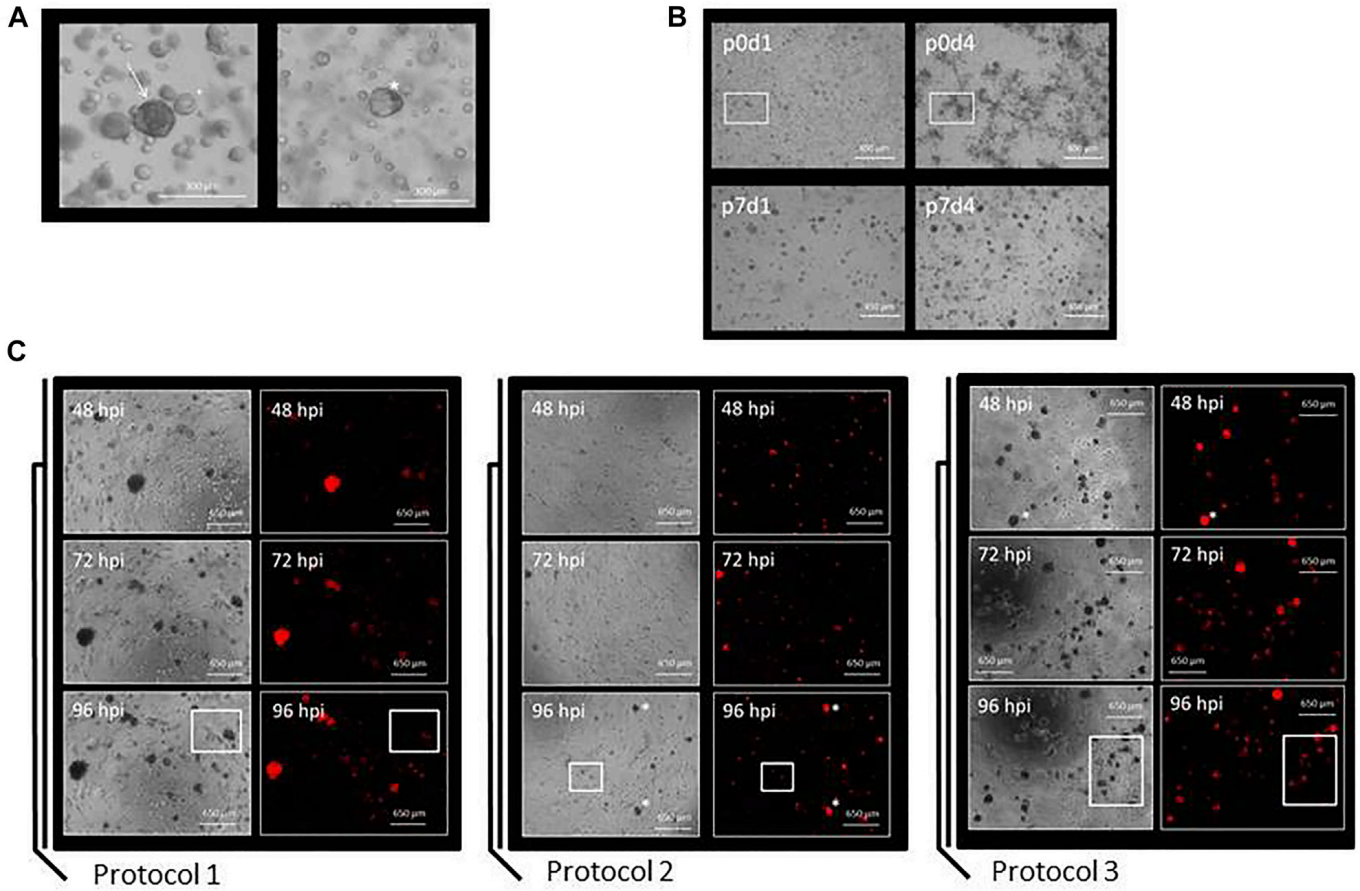
Preparation of organoid cultures from breast cancer tissues and a comparison of the three different protocols used to infect the organoid cultures with oncolytic viruses. **(A)** Organoids from breast cancer patients were mainly observed as being circular and dark colored (arrow in left panel). In some cases the organoids could be paler and appeared as more cystic structures (asterisk in right panel). **(B)** Growth characteristics of breast cancer organoid line BC-ORG 3. Pictures were taken at different passages (p) and on different days (d). Single cells being visible on day 1 (upper left panel) grew into organoids of comparable size and density during the subsequent days (other panels). **(C)** Comparison of three different protocols used for infection of the breast cancer organoid cultures. Protocol 1 was based on standard methods for two-dimensional cell cultures and the images show cells from patient sample BC-ORG 3 harvested with TrypLE and plated out in 10% Matrigel before being infected 24 h later with oncolytic virus GLV-0b347 (MOI 1) and taking phase-contrast and fluorescence pictures at different hpi. Protocol 2 involved incubating the cells immediately with the oncolytic virus and not waiting 24 h. The images show patient sample BC-ORG 5 infected with GLV-0b347 (MOI 10) before taking phase-contrast and fluorescence pictures. Protocol 3 allowed the growth of organoids and even distribution of oncolytic viruses throughout the organoids. Organoids were harvested with dispase II rather than TrypLE after being cultivated in normal growth environment without addition of oncolytic viruses. The oncolytic viruses were then added to the organoid suspension and subsequently distributed into the wells before growth of the organoids. The images show cells from patient sample BC-ORG 5 infected with GLV-0b347 (MOI 10) before taking phase-contrast and fluorescence pictures.

**FIGURE 4:**
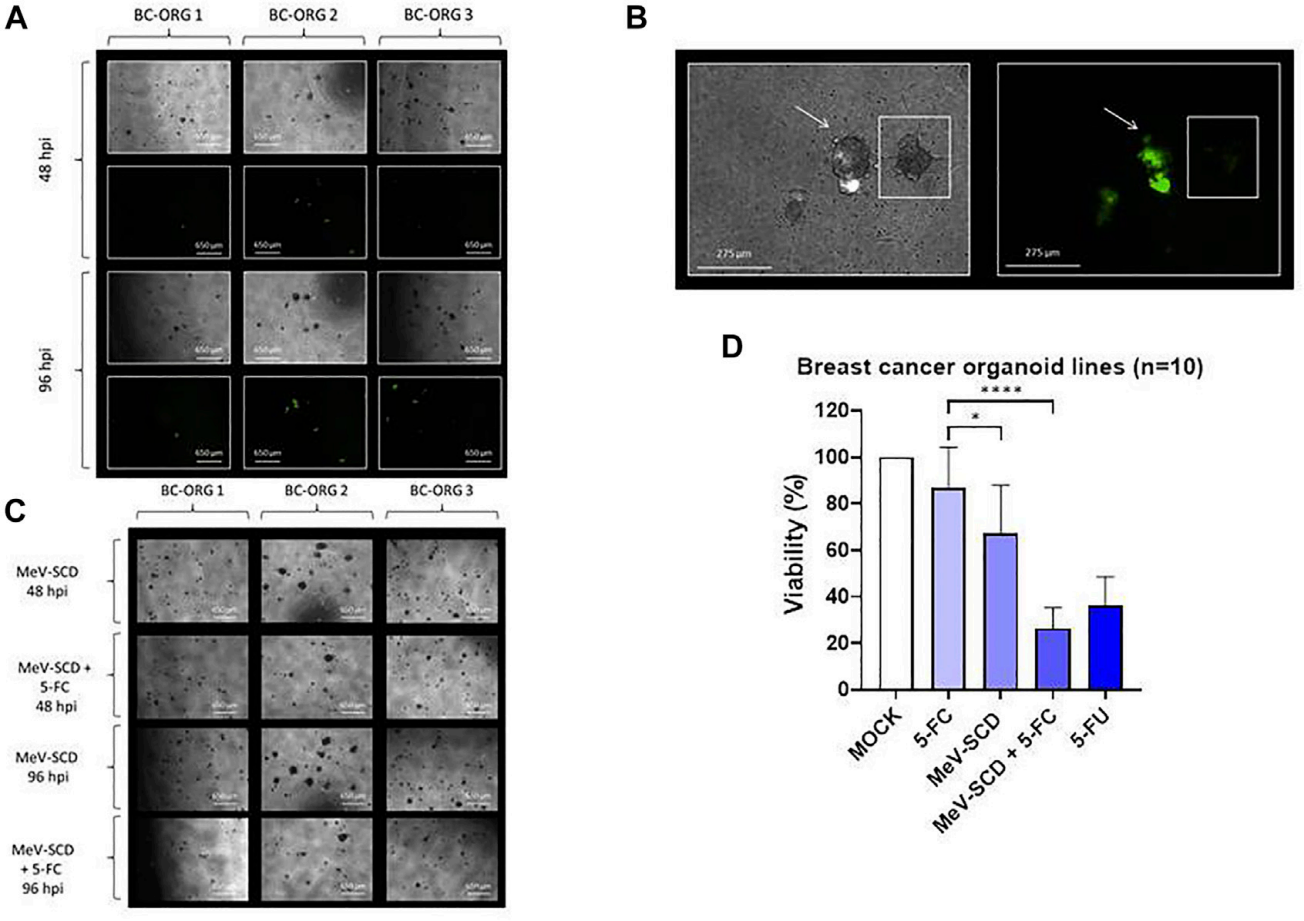
The effects of different genetically engineered oncolytic measles viruses (MeV) on organoids derived from breast cancer tissues (*n* = 10). **(A)** Phase-contrast and fluorescent images of breast cancer organoid lines BC-ORG 1, BC-ORG 2 and BC-ORG 3, respectively, at 48 and 96 hpi with MeV-GFP (MOI 10) as representative images of all 10 infected breast cancer organoid lines. **(B)** Higher magnification phase-contrast and corresponding fluorescence images of breast cancer organoid line BC-ORG 2 infected with MeV-GFP (MOI 10) at 96 hpi. The organoid highlighted with an arrow (same image in the left and right panel) has been infected with oncolytic measles virus (MeV-GFP) as seen by the green fluorescence. The neighboring organoid highlighted with a small square box (same image in the left and right panel) was not infected and did not express GFP. **(C)** Phase-contrast images of the same breast cancer organoid lines taken at 48 and 96 hpi with MeV-SCD (MOI 10) with and without 5-FC. **(D)** The effects of oncolytic MeV-SCD (MOI 10) in presence (+5-FC) and absence of 5-FC, on mean viability (%) of organoids derived from all 10 breast cancer patients (*p < 0.05, ****p < 0.0001 with post-hoc Tukey’s multiple comparisons tests). MOCK contained breast cancer organoids and culture medium.

Organoids from breast cancer patients were mainly observed as being circular and dark colored (Figure 3A, left picture). In some cases, however, the organoids could also appear paler and appeared as a more cystic structure (Figure 3A, right picture). The cells from breast cancer tissue visible on day 1 [Figure 3B, top left picture; passage 0 (p0)] over time grew into clusters and formed organoids (Figure 3B, other pictures). Tumor grade appeared to influence the growth of organoids as follows: breast cancer tissue graded G1 (organoid line BC-ORG 1) and G2 (organoid line BC-ORG 2) appeared similar and showed growth in clusters, whereas breast cancer tissue graded G3 (organoid line BC-ORG 3) displayed a more evenly distributed growth of organoids (Figure 4).

### Establishing a Reliable Protocol for Infecting the Organoid Cultures Derived From Breast Cancer Tissues With Oncolytic Viruses

In Protocol 1 we first tested the infection of breast cancer tissue organoids based on established methods for testing oncolytic virotherapy in two-dimensional cell culture models. Here the organoids were plated out in 10% Matrigel after regular passaging and infected 24 h later with GLV-0b347 (Figure 3C) and MeV-GFP (data not shown). The distribution of the resulting fluorescence was used as an indicator for the distribution of viral spread throughout the organoids. Following infections with oncolytic vaccinia virus GLV-0b347, red fluorescent organoids could be seen first at 48 h post infection (hpi) (Figure 3C, Protocol 1, upper panels) and even more fluorescent organoids could be observed at 72 hpi and 96 hpi (Figure 3C, Protocol 1, middle and lower panels). However, even at 96 hpi some organoid clusters still were not found to be infected (Figure 3C, Protocol 1, lower panels). Beyond that infections did not appear to be distributed homogeneously throughout the wells.

Hence, we set out to improve the cultivation conditions to enable both higher infection rates as well as more evenly distributed infections. For this purpose, the timing of the infections was changed. Instead of infecting organoids at 24 h after passaging, oncolytic viruses now were directly added to the organoid culture suspension containing Matrigel and breast cancer culture medium. When applying this Protocol 2 more organoids were found to be infected by GLV-0b347 at 48 hpi (Figure 3C, Protocol 2, upper panels). The viral distribution appeared to be more even (Figure 3C, Protocol 2, panels to the right) and at 96 hpi the infection was found to have spread broadly across the wells (Figure 3C, Protocol 2, lower panels). However, fewer single cells grew out into organoids when compared with Protocol 1.

The next aim was to harvest the organoids without first dissipating them into single cells. For this purpose, organoids were harvested using 100 µL of 1 mg/ml dispase II rather than TrypLE after being cultivated in normal growth environment without addition of oncolytic viruses. Oncolytic viruses were then added to the organoid suspension and seeded into the wells (Figure 3, Protocol 3). This modification resulted in a greater number of large organoids (Figure 3C, Protocol 3) when compared to both previous protocols (Figure 3C, Protocols 1 and 2). This trend could be observed at all time points, but was clearest at 96 hpi (Figure 3C, Protocol 3, lower panels). This time point also showed the greatest total amount of GLV-0b347-mediated fluorescence. As similar data was obtained with the oncolytic measles virus MeV-GFP (data not shown), Protocol 3 was utilized for all subsequent infection experiments with the oncolytic viruses of both measles and vaccinia virus origin.

### Effects of Oncolytic Measles Viruses

Employing Protocol 3, we next compared the oncolytic effects of MeV-GFP and MeV-SCD in ten organoid lines derived from 10 different breast cancer patients (Table 1). The mean values (±SD) of all 10 organoid lines are shown in Figure 4D. BC-ORG 1, BC-ORG 2 and BC-ORG 3 (Figures 4A–C) are depicted as typical representative images from all 10 breast cancer organoid lines shown in Figure 4D. All infections with MeV-GFP were successful as indicated by the green fluorescence at 48 and 96 hpi (Figure 4A; BC-ORG 1, BC-ORG 2 and BC-ORG 3).

Magnifications of phase-contrast and corresponding fluorescence images of breast cancer organoid line BC-ORG 2 taken at 96 hpi display an organoid being successfully infected with MeV-GFP (Figure 4B, highlighted by the arrows), while the neighboring organoid was found not to be infected (Figure 4B, highlighted with a square box).

Next, breast cancer organoid lines BC-ORG 1, BC-ORG 2 and BC-ORG 3 were infected with the suicide gene-enhanced vector MeV-SCD and then cultivated in presence or absence (control samples) of the prodrug 5-FC. When cultivation took place in presence of 5-FC, significantly fewer organoids could be detected (Figure 4C, compare images taken at 48 hpi [row 2 (+5-FC) versus row 1 (- 5-FC)] and at 96 hpi [row 4 (+5-FC) versus row 3 (- 5-FC)]. This effect also was quantified by employing the CellTiter-Blue^®^ Viability Assay (Figure 4D). A mock infection containing breast cancer organoids and culture medium was defined as maximum viability (100%); 1) cultivation of MeV-SCD-infected organoids in presence of the prodrug 5-FC resulted in a highly significant drop in viability to 26% (Figure 4D, bar 4) when compared to the negative control (Figure 4D, bar 2; incubation only with the non-toxic prodrug 5-FC, no infections) (*p* < 0.0001); 2) cultivation of MeV-SCD infected organoids in the absence of the prodrug 5-FC displayed an intrinsic oncolytic effect (without additional tumor cell-bound conversion of 5-FC into 5-FU and derivatives), which resulted in a significant drop of viability (*p* < 0.5) to only 67% (Figure 4D, bar 3) compared to 5-FC alone (Figure 4D, bar 2). All CellTiter-Blue^®^ Viability Assay values are expressed as the mean ± SD (n = 10).

### Effects of Oncolytic Vaccinia Viruses

**FIGURE 5:**
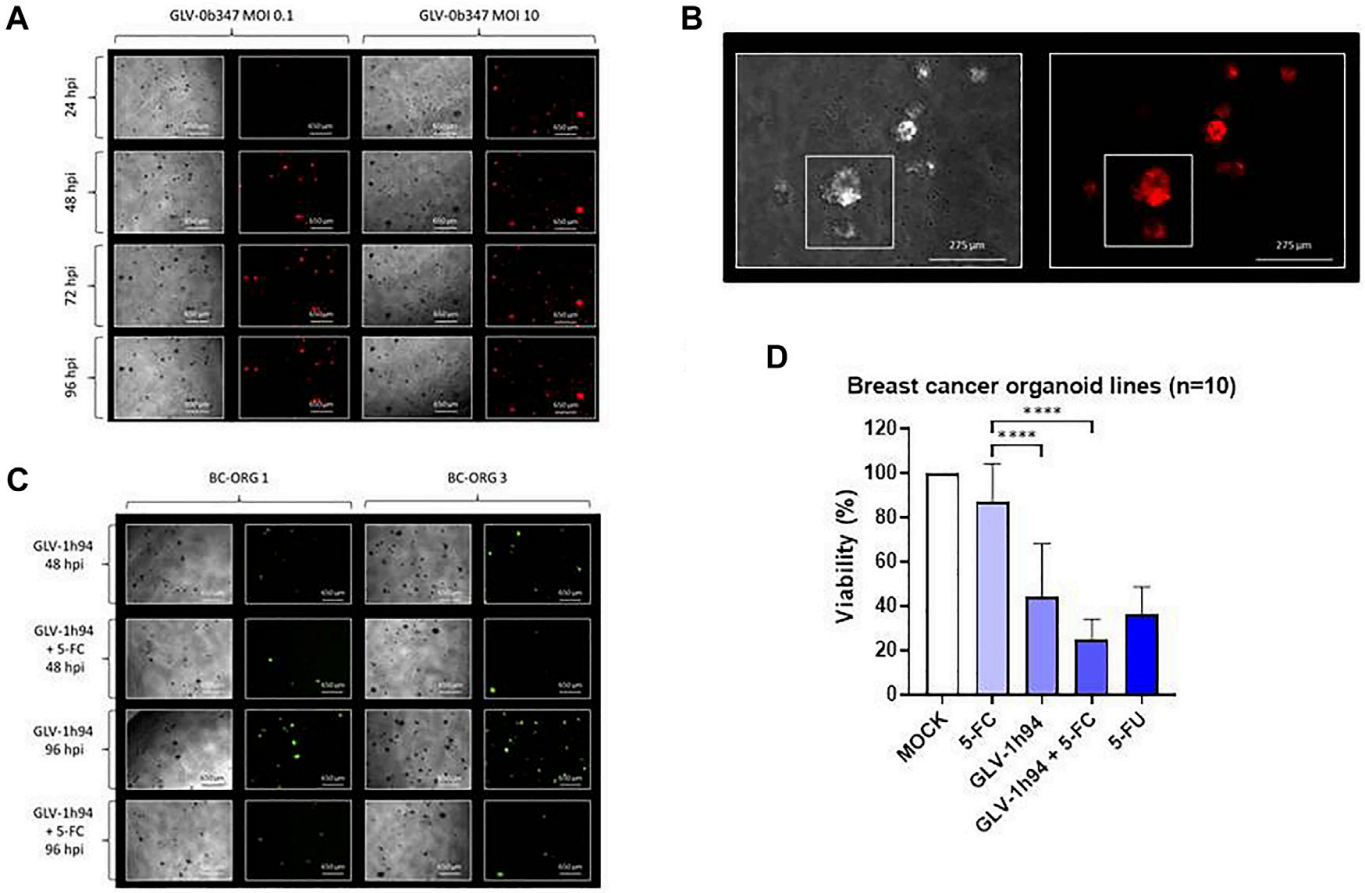
The effects of different vaccinia viruses (GLV) on organoids derived from breast cancer tissues (*n* = 10)**. (A)** Phase-contrast and fluorescent images of breast cancer organoid line BC-ORG 4 taken at 24, 48, 72 and 96 hpi with two different titers of GLV-0b347 (MOI 0.1 and 10) typically representing all 10 infected breast cancer organoid lines. **(B)** Higher magnification phase-contrast and corresponding fluorescence images of breast cancer organoid line BC-ORG 4 infected with GLV-0b347 (MOI 1), taken at 96 hpi. The images show an infected organoid with intense red fluorescence and a partially infected organoid with partial red fluorescence (see small square boxes in the panels to the left and right). **(C)** Phase-contrast and fluorescence images of breast cancer organoid lines BC-ORG 1 and BC-ORG 3 infected with GLV-1h94 (MOI 10) with and without 5-FC at 48 and 96 hpi typically representing all 10 infected breast cancer organoid lines. **(D)** The effects of oncolytic GLV-1h94 (MOI 10) in presence (+5-FC) and absence of 5-FC, on mean viability (%) of organoids derived from all 10 breast cancer patients (*p < 0.05, ****p < 0.0001 with post-hoc Tukey’s multiple comparisons tests). MOCK contained breast cancer organoids and culture medium.

We also systematically investigated the oncolytic effects of vaccinia viruses GLV-0b347 and GLV-1h94 in ten organoid lines from different breast cancer tumors, again using Protocol 3 for infection (Figure 5). We observed titer-dependent effects of GLV-0b347-mediated oncolysis which are typified by the phase-contrast and fluorescence images of breast cancer organoid line BC-ORG 4 at 24, 48, 72 and 96 hpi shown as a representation of all 10 infected breast cancer organoid lines (Figure 5A, rows 1–4). Higher magnification phase-contrast and corresponding fluorescence images of breast cancer organoid line BC-ORG 4 infected with GLV-0b347 (MOI 1) at 96 hpi showed typically infected organoids with an intense red fluorescence (Figure 5B, small square box, upper organoid) and a partially infected organoid with less intense red fluorescence (Figure 5B, small square box, lower organoid). Figure 5C shows typical phase-contrast and fluorescence images of two breast cancer organoid lines (BC-ORG 1, BC-ORG 3) at 48 and 96 hpi with the suicide gene-encoding oncolytic vaccinia virus GLV-1h94 cultivated in presence (+5-FC) and absence (- 5-FC) of the prodrug 5-FC. These images are typical of the effects seen on all 10 infected breast cancer organoid lines. The oncolytic effects of GLV-1h94 in the presence of 5-FC were found to be much stronger than in the absence of the 5-FC [Figure 5C, compare row 1 with row 2 (48 hpi time point) as well as row 3 with row 4 (96 hpi time point)].

Breast cancer organoid lines BC-ORG 1 and BC-ORG 3 were infected with suicide gene-enhanced GLV-1h94 and then cultivated in the presence or absence (control samples) of the prodrug 5-FC. Fewer viable organoids could be seen when cultivation took place with GLV-1h94 in combination with 5-FC in comparison to GLV-1h94 alone (Figure 5C compares images taken at 48 hpi [row 2 (+5-FC) versus row 1 (- 5-FC)] and at 96 hpi [row 4 (+5-FC) versus row 3 (- 5-FC)]. The CellTiter-Blue^®^ Viability Assay enabled quantification of this effect (mean ± SD) for all 10 infected breast cancer organoid lines (Figure 5D). Maximum viability was defined by a mock infection containing breast cancer organoids and culture medium (100%); 1) cultivation of GLV-1h94 infected organoids in presence of the prodrug 5-FC resulted in a highly significant drop in viability to 25% (Figure 5D, bar 4) when compared to the negative control (Figure 5D, bar 2; incubation only with the non-toxic prodrug 5-FC, no infections with oncolytic viruses) (*p* < 0.0001); 2) cultivation of GLV-1h94-infected organoids in the absence of the prodrug 5-FC displayed an intrinsic oncolytic effect (without additional tumor cell-bound conversion of 5-FC into 5-FU and derivates), which resulted in a significant drop of viability (*p* < 0.0001) to only 43% (Figure 5D, bar 3) compared to 5-FC alone (Figure 5D, bar 2).

Taken together, our results show that we have developed a protocol to assess the effects of oncolytic viruses in stable organoid cell cultures derived from breast cancer tissues. This model provides a promising *in vitro* method to help further testing and engineering of new generations of virotherapeutic vectors for clinical applications. Beyond that we are opening the way for a future personalized pretesting and treatment of breast cancer patients with oncolytic viruses.

## Discussion

In this study we utilized an established experimental model of stable organoid cell cultures from breast cancer tissue first described and characterized by Sachs et al. (Sachs et al., 2018) to assess the effects of genetically engineered oncolytic viruses using two different types of oncolytic viruses, i.e., measles vaccine viruses and vaccinia viruses. Experimental investigation of personalized treatment of breast cancer patients requires a reliable patient-derived breast cancer model. This will facilitate the transfer of treatment options from bench to bedside.

Currently established models for breast cancer research include immortalized human tumor cell lines, rodent xenografts, and immunodeficient, xenograft mouse models (Bosma and Carroll, 1991; Kamb, 2005; Drost and Clevers, 2018). These models have major disadvantages and may only insufficiently represent the patterns of the original cancer patient tumor tissues (Bosma and Carroll, 1991; Kamb, 2005). In contrast, a three-dimensional organoid model based on patient-derived tumor samples should be able to recapitulate the structure of the original tumor and capture disease heterogeneity and the characteristics of the patient’s individual tumor (Drost and Clevers, 2018; Sachs et al., 2018). Breast cancer organoid lines were successfully and reproducibly established from different patients in this study and several different tumor samples were used to capture the heterogeneity of breast cancer tissues.

The goal of the study was to establish a protocol for assessing oncolytic therapy in organoid cultures derived from breast cancer patients. All protocols used in this work showed success of oncolytic virotherapy as demonstrated by detection of fluorescence by microscopic images and reduction of organoid viabilities, measured with the CellTiter-Blue^®^ Viability Assay. To improve viral spread, the time-point of infection was varied in relation to the passaging process to allow infection of organoids in a three-dimensional setup rather than aiming at single cells being infected in a three-dimensional setup. This resulted in a more homogenous distribution of the viral infections und enhanced the reduction of organoid viabilities through virus-mediated oncolysis. The CellTiter-Blue^®^ Viability Assay was able to measure the reduction of organoid viabilities. However, it was important to note that Matrigel exhibits an inherent background signal in this assay and therefore needs to be corrected for when performing such measurements. We then set out to test whether single cells contained in the organoid setup interfere with the measurements *via* the CellTiter-Blue^®^ Viability Assay.

At this step of our protocol development, cultivation of the breast cancer organoid lines did not yet include immune cells. For example, measles viruses normally induce an IFN response which triggers an immune response directed against the tumor cells (Krabbe and Altomonte, 2018). Tumor cells are known for mutations in IFN signaling thereby enabling an enhanced spread of oncolytic viruses throughout the tumor which also facilitates a subsequent anti-tumoral immune response (Kirn et al., 2001). The cultivation of breast cancer organoids without immune cells does not allow a measurement of the effects this immune response on the oncolytic virotherapy. Dijkstra *et al.* (Dijkstra et al., 2018) successfully incorporated autologous peripheral blood monocytes derived from patient blood samples into the corresponding colon and lung cancer organoid setup. In this setting, the T-cell infiltration of the patient’s cancer organoids could be measured and displayed an efficient killing of cancer organoids. Accordingly, the addition of patient-derived peripheral blood monocytes to our breast cancer organoid setup could be a next step to improve our model yet further. It would allow a more accurate representation of the environment surrounding the tumor in the patient. The addition of these cells to oncolytic virotherapy also would allow a better assessment of the importance of the immune response on the efficiency of the oncolytic virotherapy.

The incorporation of immune cells into the organoid cultivation would also allow the evaluation of oncolytic viruses that have been engineered for triggering an immune response against tumor cells specifically. For example, talimogene laherparepvec/T-VEC constitutes the first clinically licensed (for advanced malignant melanomas) oncolytic virus (IMLYGIC^®^) in the category of therapeutically armed oncolytic viruses. T-VEC is based on a recombinant herpes-simplex virus 1 (Hu et al., 2006) and also interferes with the IFN pathway resulting in enhanced tumor selectivity and effectivity (Liu et al., 2003). An additional genetic modification results in a higher production of class I major histocompatibility complex (MHC) molecules important for triggering an immune response against the host cells (Hill et al., 1994; Hill et al., 1995). The viral genome has been genetically modified to include the arming with a granulocyte-macrophage colony stimulating factor (GM-CSF) gene (Greig, 2016). Also T-VEC needs to be compared to other oncolytic viruses in our organoid model in future work.

Oncolytic viruses can be classified into three different types; 1) oncolytic viruses with natural anti-neoplastic properties, 2) oncolytic viruses designed for tumor-selective replication, and 3) armed oncolytic viruses (Hartkopf et al., 2011). Four oncolytic viruses derived from two virus families were tested in our organoid model. Two viruses exhibiting an intrinsic oncolytic activity (MeV-GFP, GLV-0b347) were used as well as the same two virus backbones additionally being armed with suicide genes to enhance naturally occurring oncolytic activity (MeV-SCD, GLV-1h94), thereby achieving an additional tumor-cell bound conversion of 5-FC into 5-FU and derivatives, i.e., a tumor cell-localized chemotherapy.

Measles viruses and Vaccinia viruses show innate oncolytic potential (Hartkopf et al., 2011). MeV-GFP and GLV-0b347 are recombinant vaccine viruses in which the marker genes 1) green fluorescent protein (MeV-GFP) or 2) red fluorescent protein (GLV-0b347) are encoded as transgenes. The insertion of the SCD/FCU1 suicide gene to the measles virus genome allowed evaluation of the additional influence of such a suicide gene on organoid viability. MeV-GFP and MeV-SCD showed a comparable reduction of organoid viability. Of note, the effect of MeV-SCD could be enhanced significantly when the prodrug 5-FC was added to the culture medium. Under this condition, organoid viabilities displayed a similar drop in organoid viability as when treated with the chemotherapeutic compound 5-FU. These results demonstrate that wild-type vaccine measles virus exerts an intrinsic oncolytic effect in the breast cancer organoid lines. This basic oncolytic effect of the measles virus is further enhanced when it encodes a suicide gene which in presence of the prodrug 5-FC conveys an additional tumor cell-localized chemotherapy.

GLV-0b347 and GLV-1h94 are both vaccinia viruses, yet include a different genetic backbone. GLV-0b347 is based on a *Western Reserve* strain backbone and GLV-1h94 on a *Lister* strain backbone. As previous research had demonstrated different distribution rates of the viruses in different types of tissues, vaccinia viruses with different backbones were used for the experiments (Zhang et al., 2007). However, the reduction in organoid viability between GLV-0b347 and GLV-1h94 was comparable. This suggests similar distribution of both vaccinia viruses in breast cancer tissues. Both, GLV-0b347 and GLV-1h94 also inhibited cell viability in breast cancer organoid lines. As expected, oncolytic effects of GLV-0b347 were found to be dependent on the virus titer used. The oncolytic effects of both MeV-SCD and GLV-1h94 were enhanced in the presence of the prodrug 5-FC which is converted to the active and cytotoxic metabolite 5-FU and 5-fluorouridine by the expressed suicide gene conversion enzyme SCD/FCU1.

The addition of 5-FC to the infection with GLV-1h94 led to a significant decrease in organoid viability in comparison to the infection with vaccinia viruses alone or 5-FC alone. Importantly, the combination of these agents resulted in greater reduction in organoid viability than 5-FU alone when using the same compound concentrations (1 mmol/L each). Therefore, the oncolytic effect of vaccinia viruses equipped with the suicide gene and the prodrug 5-FC cannot be based solely on the effects of the production of 5-FU. The oncolytic effect of the GLV-1h94 resulted in an additional reduction of organoid viability. The innate oncolytic effect of vaccinia viruses as well as the combination with a suicide gene seemed to work synergistically.

Taken together, our organoid model enabled oncolytic viral infection in breast cancer organoid lines. The next step would be to compare and contrast the effects of other oncolytic viruses such as T-VEC and test them on our breast cancer organoids to establish a panel most likely to be effective for oncolytic virotherapy of breast cancer. The viruses included in this panel should combine different approaches such as viruses with naturally occurring oncolytic potential, genetically modified virus for tumor selectivity and armed oncolytic viruses for enhanced cell killing or enhanced triggering of an immune response. Our study showed that infections with oncolytic viruses are possible in our organoid culture setup of primary breast cancers.

## Conclusion

This study shows that it was possible to develop a protocol that could be used to assess the effects of two different oncolytic viruses on cell viability in established patient-derived organoid cell cultures from breast cancer tissue. The greatest oncolytic effects were observed for oncolytic viruses engineered to express a suicide gene (MeV-SCD; GLV-1h94) in the presence of the prodrug 5-FC. Thus the model provides a promising *in vitro* method for investigating the effects of different oncolytic viruses for treating breast cancer, thereby facilitating the correlation to *in vivo* results.

## Data Availability

The raw data supporting the conclusions of this article will be made available by the authors, without undue reservation.
